# GH and the cardiovascular system: an update on a topic at heart

**DOI:** 10.1007/s12020-014-0327-6

**Published:** 2014-06-28

**Authors:** Jörgen Isgaard, Michele Arcopinto, Kristjan Karason, Antonio Cittadini

**Affiliations:** 1 Laboratory of Experimental Endocrinology, Department of Internal Medicine, Sahlgrenska Academy, University of Gothenburg, Gröna Stråket 8, 413 45 Göteborg, Sweden; 2 Department of Internal Medicine, Cardiovascular and Immunological Sciences, Federico II University, Naples, Italy; 3 Department of Cardiology, Sahlgrenska University Hospital, Göteborg, Sweden

**Keywords:** Growth hormone, Insulin-like growth factor, Cardiovascular, Heart

## Abstract

In this review, the importance of growth hormone (GH) for the maintenance of normal cardiac function in adult life is discussed. Physiological effects of GH and underlying mechanisms for interactions between GH and insulin-like growth factor I (IGF-I) and the cardiovascular system are covered as well as the cardiac dysfunction caused both by GH excess (acromegaly) and by GH deficiency in adult hypopituitary patients. In both acromegaly and adult GH deficiency, there is also increased cardiovascular morbidity and mortality possibly linked to aberrations in GH status. Finally, the status of the GH/IGF-I system in relation to heart failure and the potential of GH as a therapeutic tool in the treatment of heart failure are reviewed in this article.

## Introduction

Besides well-recognized effects such as promoting longitudinal bone growth in childhood and exerting a number of metabolic effects, growth hormone (GH) has an important role for the development of a normal heart [1]. GH also has a major impact on maintaining the structure and function of the normal adult heart [1, 2]. Apart from stimulating cardiac growth and possibly also contractility, GH/IGF-I interacts with the vascular system and has a role in the regulation of vascular tone and thereby peripheral resistance. Also central effects including modulation of sympathetic outflow contribute to regulation of peripheral resistance [3].

The myocardium and vessels express IGF-I [4–6] and functional receptors for both GH [7–9] and IGF-I [10, 11], and IGF-I production is up-regulated in response to GH [5]. Thus, there are possibilities of direct actions of GH as well as endocrine or autocrine/paracrine effects of IGF-I on the cardiovascular system. However, although interaction of the GH/IGF-I axis and the cardiovascular system has been extensively studied, the relative importance of direct effects of GH and local and endocrine IGF-I remains unclear.

## Vascular effects of GH and IGF-I

### Regulation of peripheral resistance

Both GH and IGF-I have been suggested to have a regulatory role for peripheral resistance, although it is often difficult to differentiate between direct effects of GH and effects mediated by IGF-I. Evidence to suggest rapid vasoactive effects of IGF-I includes the finding that intravenous administration of IGF-I decreases mean arterial blood pressure within a few minutes in normal rats [12, 13]. In a human study, stroke volume and cardiac output were increased, but blood pressure unchanged a few hours after a single injection of IGF-I [14]. Moreover, in patients with chronic heart failure (CHF), IGF-I infusion increased cardiac output and decreased peripheral resistance within a time span of 2 h [15]. A similar effect of GH was seen after 24 h when serum IGF-I was concomitantly increased [16]. These data suggest that IGF-I has potent, acute functional effects on the cardiovascular system. In a more recent study, it was shown in healthy subjects that GH directly increased forearm blood flow paralleled by a decrease in peripheral resistance [17]. Moreover, these changes were suggested to be mediated by stimulation of endothelial function through the NO system. However, the effect was not seen until 4 h after GH infusion, and it is possible that GH actions were mediated by local production of IGF-I in the periphery.

Despite accumulating evidence supporting the vasodilating effects of IGF-I, there have only been a few studies addressing the role of IGF-I in more long-term, physiological regulation of peripheral resistance. Lembo et al. [18] showed that mice with a mutant IGF-I allele and 30 % of wild-type IGF-I levels present in all tissues, and serum have elevated blood pressure. In analogy, transgenic mice with a liver-specific knock-out of IGF-I and an 80 % decrease of circulating IGF-I also display a significant elevation of blood pressure, indicating that a decrease in endocrine-acting IGF-I results in an elevation of blood pressure [19].

Several studies suggest that the vascular actions of IGF-I may be mediated through release of NO and/or other vasodilators from the endothelium. IGF-I stimulates NO release from cultured endothelial cells [20], vascular smooth muscle cells [21] as well as aortic preparations [13]. Pre-treatment with the NO-synthase inhibitor L-NAME is capable of abolishing the vasodilatory effect of IGF-I in large arteries [12, 22–24]. There are also reports of eicosanoids as mediators of the vasodilating effects of IGF-I and that indomethacin may prevent vasodilatation by IGF-I [22, 24, 25]. Moreover, IGF-I may cause vasorelaxation through non-endothelium-dependent actions [26], possibly by increasing the activity of the Na^+^, K^+^-ATPase in vascular smooth muscle cells [27]. Another possible mechanism for the GH/IGF-I influence on vascular tone involves the regulation of gene expression of the vascular smooth muscle K_ATP_ channel [28]. This ATP sensitive potassium channel consists of two subunits, the inwardly rectifying potassium channel Kir6^.^1 and the sulfonylurea receptor 2B (SUR2B) [29] where Kir6^.^1 has been proposed to be critical for regulation of vascular tone [30]. Moreover, the smooth muscle K_ATP_ channel is also the target for the anti-hypertensive potassium channel opening drugs [29]. GH treatment of hypophysectomized (hx) rats resulted in increased mRNA levels of both Kir6^.^1 and SUR2B, and this was correlated to a lowering of systolic blood pressure [28].

### Central effects

It has also been suggested that some vasoactive effects of GH may have central origin. In a comparison between GH-deficient (GHD) patients without GH substitution and healthy controls matched for BMI, it was shown that GHD patients had markedly increased muscle sympathetic nerve activity [3]. Moreover, 1 year of substitution therapy with GH had a modest but significant effect on decreasing sympathetic nerve activity to the muscle vascular bed [31]. This could suggest that GH may regulate the central sympathetic outflow affecting peripheral resistance.

### Vascular consequences of GHD

Conflicting results regarding blood pressure and peripheral resistance have been reported in the literature, although it appears that at least in certain patients, GH treatment can lower peripheral resistance. In GH-deficient adults without GH replacement therapy, an increased prevalence of hypertension was reported in a large study that mainly consisted of adult-onset GH-deficient patients [32]. However, unchanged blood pressure has also been reported [33] and in studies mainly consisting of young GH-deficient adults, even reduced blood pressure has been reported [34–36]. In another study, GH replacement therapy did not affect systolic blood pressure, whereas diastolic blood pressure was decreased due to reduced peripheral vascular resistance [37]. Also in men with the metabolic syndrome but without severe GH deficiency, a lowering of diastolic blood pressure was observed after GH treatment for 9-months [38]. A stimulatory effect of GH on nitric oxide production [39] could possibly explain the reduced peripheral vascular resistance and the reduction in diastolic blood pressure in response to GH supplementation that have been observed in some trials. However, there are also studies showing unchanged [40] or even increased [41] blood pressure during GH replacement. Taken together, it can be speculated that GH replacement reduces blood pressure only in subgroups of GH-deficient adults, possibly in patients with high base-line diastolic blood pressure [42], such as GH-deficient patients with previous Cushing´s disease or elderly GH-deficient adults.

Atherosclerosis has also been observed in hypopituitary adults without GH replacement therapy. In a study by Markussis et al. [33], increased carotid artery wall thickness was observed in GH-deficient adults. The results of several later studies suggest that GH replacement may reverse early atherosclerotic changes in the carotid arteries in GH-deficient adults [43, 44].

### Vasculature in GH excess

It would appear that more short-term exposure to high levels of GH leads to a decrease of peripheral resistance. Some pioneering experimental studies focusing on the hemodynamic effects of chronic GH hypersecretion employed a rat model with an implantable GH-secreting tumor was used as an experimental model [45]. These rats displayed cardiomegaly and increased cardiac contractility and output, while peripheral resistance was decreased [45]. In human acromegalics, three stages of cardiovascular disease have been identified—an early, intermediate and late stage of the disease [1]. Interestingly, although rarely diagnosed in the early stage, patients with only a relatively short duration of acromegaly display a “hyperkinetic” cardiovascular system with increased cardiac output and decreased total peripheral resistance [1]. However, if the disease is not treated, it progresses into more advanced stages with hypertension as a common finding. Hypertension has been reported in the bGH mouse model [46] and in humans, studies suggest a prevalence of hypertension of 20–50 % in acromegalic patients [47]. The mechanisms for the increased prevalence of hypertension have been suggested to include an expansion of plasma volume, stimulation of smooth muscle cell growth leading to increased vascular resistance, and increased insulin resistance as a potential facilitator of increased blood pressure (for review see: [48]).

The prevalence of atherosclerosis in acromegalic patients is controversial. However, recent studies suggest that acromegalic patients do not have increased prevalence of coronary artery disease [49, 50], carotid atherosclerosis, or carotid internal media thickness compared to normal subjects [49, 51]. In a recent European study on 200 acromegalic patients matched with a control group, it was reported that patients with active acromegaly had significantly lower levels of high-sensitive C-reactive protein (hs-CRP) both compared with patients with controlled acromegaly and with a matched reference population [52]. Possibly, a lower hs-CRP may be linked to lower than expected prevalence of atherosclerosis in patients with active acromegaly, despite the presence of other risk factors such as insulin resistance and hypertension.

## Effects of GH and IGF-I on cardiac structure

### Growth and development

It is generally difficult to demonstrate in vitro effects of GH, and consequently, many studies have failed to show direct, IGF-I-independent hypertrophic effects of GH on cardiomyocytes [53, 54]. However, it has been suggested that GH may cause alterations in cardiomyocyte metabolism and stimulate cardiac growth independently of IGF-I [55, 56]. When performing in vitro studies, it is easier to see effects of IGF-I compared to GH, and many studies have shown that IGF-I increases protein synthesis [53, 57] and the size of cardiomyocytes [54] in vitro.

Several genes have been identified as targets of GH and IGF-I, and it has previously been shown that IGF-I stimulates the expression of muscle-specific genes in rat cardiomyocytes [54]. In hx rats, GH and IGF-I treatment stimulated similar cardiac gene expression (skeletal a-actin, atrial natriuretic factor) but neither IGF-I nor GH caused a shift of myosin heavy chain isoforms [58]. Interestingly, it has been shown that expression of activated calcineurin mimics the hypertrophic effects of IGF-I in skeletal muscle [59].

Cardiac hypertrophy requires concomitant remodeling of the heart, and attention has been focused on the role of the extracellular matrix in the remodeling process [60]. It has been shown that IGF-I promotes collagen synthesis by fibroblasts [61] and that GH increases the collagen deposition rate in the heart [62], while in GH-induced hypertrophy, the volume fraction of collagen is normal [63, 64]. Thus, available data are in line with an increased synthesis as well as breakdown of extracellular matrix with a finally unchanged cardiac collagen concentration by GH.

Besides obvious growth promoting effects on cardiomyocytes, GH and IGF-I may also modulate the structure of the myocardium by preventing cardiomyocyte loss through apoptosis. Ongoing apoptosis has been demonstrated also in the normal heart [65], and there is substantial evidence that IGF-I acts as an inhibitor of apoptosis. In line with this, it has been proposed that an anti-apoptotic effect GH/IGF-I may serve to protect the myocardium in conditions with ischemic injury [66, 67]. However, although an increased number of nuclei in the heart of GH-treated adult rats have been reported [64], there is still no evidence that GH or IGF-I can affect cardiomyocyte number in the normal heart unless overexpressed from the embryonic cell stage [68].

### Cardiac structure in GHD

A number of studies have addressed cardiac structure in patients lacking GH. It has been reported that GH-deficient adults without GH replacement therapy have reduced left ventricular mass and cardiac output [34] and decreased exercise capacity [1]. The results of some studies suggest that the cardiac dysfunction may be more severe in GH-deficient patients with childhood-onset disease than in patients with adult-onset disease due to the lack of GH/IGF-I during growth and development of the heart [34]. In a study by Longobardi et al. [69], however, patients with childhood- and adult-onset disease younger than 40 years of age were studied using equilibrium radionucleotide angiography. In this study, left ventricular ejection fraction was decreased by 17 % at rest and 29 % at peak exercise as compared with age- and sex-matched controls without any difference between childhood- and adult-onset disease [69].

Short-term and placebo-controlled studies have shown that GH replacement therapy in adult GH-deficient patients has an anabolic effect on cardiac structure [35, 37, 70], resulting in an improvement in both diastolic [71], and systolic [35, 37, 70, 72] function. A few open label studies have determined the long-term effects of GH replacement therapy on cardiac function. Thirty-eight months of GH replacement therapy normalized cardiac structure, whereas heart rate and cardiac index increased to supranormal levels [73]. In another study, including 38 young men with childhood-onset GH deficiency, GH replacement therapy for 55 months (range 39–69) increased stroke volume and maximal exercise capacity without any long-term increase in left ventricular mass [41].

In 7 adults with adult-onset GH deficiency, 42 months of open GH treatment increased the left ventricular mass and decreased the atrial emptying index, which reflects diastolic function, as compared with healthy matched controls [74]. The results of this last study might suggest, although a higher dose of GH was given than that used today, that increased age might increase the susceptibility to develop inappropriate increment in left ventricular mass during long-term GH replacement therapy. Therefore, studies so far suggest that low dose, individualized GH replacement therapy improves cardiac function with less risk of developing cardiac hypertrophy. This may explain, combined with the increased muscle strength, the improved exercise capacity in GH-deficient adults observed after GH replacement. However, if an inappropriately high dose of GH is given, there is a risk of an unwanted increment in left ventricular mass, particularly in elderly GH-deficient patients during long-term treatment.

### Cardiac structure in acromegaly

Acromegaly is usually a slowly progressing disease where gradually signs of changes in cardiac structure and function develop in a considerable number of patients. Advanced acromegalic cardiomyopathy is characterized by cardiomegaly, ventricular hypertrophy, replacement fibrosis, and degeneration of cardiomyocytes [47, 75]. The precise mechanism for this gradual decline in cardiac structure is not known. However, transgenic mice overexpressing bGH were also found to have deterioration in myocardial bioenergetics that was linked to ultrastructural changes in mitochondria and depression of systolic function [76].

Treatment strategies for acromegaly include surgery and pharmacological options, mainly with long-acting somatostatin analogs, a GH-antagonist and dopamine agonists. In therapy resistant cases, radiotherapy may also be an additional option. It has been reported that both surgery and pharmacological treatments with a decrease in GH and IGF-I levels lead to improvement in cardiac structure and function (for review see [48].

## GH/IGF-I and contractility

The notion of GH and IGF-I as molecules endowed with stimulatory properties on myocardial contractility is interesting but so far, only demonstrated in experimental studies. In line with reports on cardiac hypertrophy, there are several in vitro studies demonstrating direct effects of IGF-I on intrinsic cardiac contractility [77–79], while there is still so far no evidence of direct, IGF-I-independent effects of GH on cardiac contractility. However, if animals are treated with GH in vivo, allowing stimulation of IGF-I synthesis, subsequent in vitro assessment shows improved contractility [80, 81]. Accordingly, decreased contractility has been demonstrated in dwarf rats with GH/IGF-I deficiency [82–84]. In contrast, a paradoxical enhancement of cardiac contractility was observed in the IGF-I mutant mouse [18].

At least three different mechanisms have been suggested for the GH/IGF-I to induce increased cardiac contractility: 1. altered intracellular Ca^2+^ transients, 2. increased sensitivity of myofilaments to Ca^2+^, and 3. a shift in myosin isoforms.

Regarding intracellular Ca-transients, IGF-I has been shown to acutely affect Ca^2+^ currents within the cardiomyocyte, with increased peak Ca^2+^ levels [78, 85] and an altered time course of the current [78] in association with increased contractility. Specifically, the activity of L-type Ca^2+^-channels was acutely increased by IGF-I in vitro [86]. In cardiomyocytes from acromegalic rats, the action potential duration was increased due to a decrease in density of a transient outward current carried by K^+^, which, in turn prolongs the Ca^2+^-influx through L-type Ca^2+^-channels [87]. In contrast to other in vitro studies, the acute increase of inotropy by IGF-I was associated with decreased peak Ca^2+^ levels but increased Ca^2+^ sensitivity of the contractile elements in isolated whole heart preparations [79]. No influence of GH on Ca^2+^ currents has been seen in acute settings [79, 85], while after more long-term treatment in vivo, GH has been suggested to increase peak intracellular Ca^2+^ levels measured ex vivo [80, 81]. Accordingly, reduced peak intracellular Ca^2+^ levels as well as slowed intracellular Ca^2+^-clearing have been demonstrated in GH/IGF-I deficiency [84], while others report peak intracellular Ca^2+^ levels to be unchanged in GH/IGF-I deficiency [83].

To date, little has been known about possible gene regulations involved in the action of GH/IGF-I in altering Ca^2+^ handling. An up-regulation of sarcoplasmic reticulum ATPase (SERCA) levels has been suggested to contribute to the increased contractile function elicited by GH after myocardial infarction [81] and in rapid pacing heart failure [88], while another study [89] has not been able to detect any change in SERCA expression. SERCA may increase contractility by enhancement of the so-called contractile reserve, i.e., the Ca^2+^ storage within the sarcoplasmic reticulum, allowing higher peak Ca^2+^ levels upon stimulation. Ueyama et al. [90] also suggested that GH treatment in cardiomyopathic, but not normal, hamsters preserved cardiac ryanodine receptor density.

*Myofilament Ca* ^*2*+^-*sensitivity and myosin isoform shift*. GH/IGF-I has been suggested to increase myofilament Ca^2+^ sensitivity [79, 84, 91] and maximum Ca^2+^ activated force [79, 80, 91]. However, data are conflicting, and others report unchanged [85] or even decreased [80] myofilament Ca^2+^ sensitivity by GH/IGF-I. In dwarf rats, unchanged myofilament Ca^2+^ sensitivity has been reported [83, 84], although maximum Ca^2+^ activated tension was less [83]. In animal models of GH excess, a shift toward a myosin isoform with lower ATPase activity has been demonstrated, which may decrease the energy demand of the contractile process [91, 92].

Taken together, available data suggest that GH/IGF-I may increase cardiac contractility through modulations of intracellular Ca^2+^ transients, myofilament Ca^2+^ sensitivity, and myosin isoform expression, although the findings depend upon different experimental settings and among studies. Besides regulation of ion channel activity, GH/IGF-I may also regulate the expression of ion channels. Solid evidence for increased contractility by GH and IGF-I is still lacking.

## Cardiovascular aspects of GHD

### Cardiovascular risk factors

In addition to the cardiac dysfunction, several cardiovascular risk factors are associated with adult GH deficiency. These include insulin resistance [93, 94], unfavorable alterations in serum lipid pattern including increased serum low density lipoprotein (LDL)-cholesterol concentration [95], decreased fibrinolysis [96], and increased sympathetic nervous activity [3]. Other cardiovascular risk factors beneficially affected by GH treatment include homocysteine and C-reactive protein (for review see: [97]). In addition, pregnancy-associated plasma protein-A (PAPP-A) has been found to be elevated in GHD patients [98]. This may be of particular interest, since PAPP-A is both a cardiovascular risk factor and a mediator of IGF-I bioavailability [98]. Body composition has a tendency to deteriorate in hypopituitary adults with increased body fat and decreased body cell mass [99]. Furthermore, extracellular water is decreased, [32], which may result in reduced preload of the heart (Starling effect). Finally, decreased sweating, impaired thermoregulation, and increased risk for developing hyperthermia during exercise in hot environments [100] have been observed in GH-deficient adult patients.

GH replacement therapy normalizes most of the cardiovascular risk factors observed in hypopituitary patients [101]. Body composition is rapidly normalized by GH replacement therapy [95] and appears to be partly sustained after 15 years of therapy [102]. Aberrations in serum lipid concentrations and fibrinolysis are also improved by GH replacement therapy [95, 103]. A modest improvement of sympathetic nerve activity was found after 1 year of GH replacement [31]. The effects by GH replacement on insulin sensitivity are still controversial [104, 105], although in one study, 7-year GH replacement provided protection from the age-related decline in insulin sensitivity [105].

### Morbidity and mortality

A landmark retrospective study by Rosén and Bengtsson [32] showed doubled overall mortality due to increased cardiovascular mortality in hypopituitary adults, as compared with the normal population. The hypopituitary patients in the study by Rosén and Bengtsson [32] had routine hormonal replacement therapy which did not include GH at that time. Two additional retrospective studies [106, 107], and one prospective study [108], have confirmed that the increase in total mortality in hypopituitary adults without GH replacement therapy is due to increased cardiovascular mortality. A more recent study was based on data recorded by the Board of Health and Welfare in Sweden [109]. This study confirmed an increased overall mortality in 1,411 hypopituitary patients without GH replacement therapy [109]. The increase in the total number of myocardial infarctions (fatal and non-fatal) was less marked than the increase in cerebrovascular events in this study [109]. The risk ratio for myocardial infarction in the hypopituitary patients without GH replacement therapy was 1.40, 95 % CI 1.10–1.75.

There is no abundance of data regarding the effect of GH replacement therapy on cardiovascular morbidity and mortality. In the report by Svensson et al. [109], there was, however, also a prospective study of 289 hypopituitary patients that received GH replacement therapy at a single center. The mean duration of GH treatment was 60 months (range 2–118 months). It was demonstrated that the risk ratio for myocardial infarctions was even lower in the 289 hypopituitary patients on GH replacement therapy than in the general population. Taking into account that the relative risk of myocardial infarctions was increased in hypopituitary patients without GH replacement, the reduced rate of myocardial infarctions in hypopituitary on GH replacement therapy clearly indicates that GH replacement therapy will be effective in preventing myocardial infarctions in hypopituitary adults. In a recent prospective observational study on GH-deficient adults from the US on 1988 GH-treated patients and 442 untreated and a mean follow-up for 2.3 years, there was no increase in overall mortality rate or cardiovascular events between the two studied groups [110]. However, the follow-up was comparatively short which makes conclusion on long-term effects uncertain.

## A potential therapeutic role for GH in heart failure?

Despite considerable advances in both medication strategies and use of medical devices in patients with heart failure in the last decades, the prognosis is still poor, and there is a continued interest to develop alternative or additional treatment modalities.

One of the first clinical publications to mention possible beneficial effects of GH in heart failure was the paper by Caidahl et al. [37] in 1994, describing improvement in systolic function in GH-deficient patients treated with GH.

This triggered several research groups to study effects of GH and/or IGF-I in experimental models with states of impaired cardiac function. In an established rat model of congestive heart failure following ligation of the left coronary artery, GH and IGF-I have been found to increase stroke volume and cardiac output [111, 112], also in the presence of ACE inhibition [113]. GH treatment of rats with experimental myocardial infarction has also been found to improve myocardial bioenergetics [114] and long-term survival [115].

The first clinical studies regarding GH treatment in heart failure were limited to case reports [116, 117] where GH administration dramatically improved cardiac function. A subsequent small open study of seven patients with idiopathic dilated cardiomyopathy and CHF without GH deficiency, who received GH treatment for 3 months, demonstrated considerable improvement of left ventricular ejection fraction, cardiac output, and exercise performance [118]. Further studies have demonstrated beneficial effects in patients with CHF due to both ischemic and idiopathic dilated cardiomyopathy with improvements in hemodynamics when GH was added both as a maintenance therapy and as short-term infusion [119, 120]. Moreover, another interesting observation was that patients with a low base-line serum IGF-I had less beneficial effects of an acute GH infusion [121]. A later study showed that GH treatment decreased circulating levels of cytokines such as TNF-α and IL-6 and apoptotic agents such as FAS and its soluble ligand in patients with heart failure [122].

Even though a more recent placebo-controlled trial on 16 patients with CHF showed correction of endothelial dysfunction and improved non-endothelium dependent vasodilation [123], other randomized, placebo-controlled studies have failed so far to show any significant GH-mediated improvement of cardiac performance in patients with heart failure, despite significant increases in IGF-I [124, 125]. However, in a follow-up study of the same patients in the former study, analyzed in more depth, Perrot et al. [126], found a significant increase in left ventricular mass, which correlated with serum IGF-I. Moreover, there was a significant increase of ejection fraction in those patients that responded with higher serum IGF-I levels during GH treatment. Acevedo et al. [127] performed a randomized controlled trial of 19 patients with daily GH administration for 8 weeks. However, at the end of treatment, there was no significant effect of GH on LVEF or peak oxygen consumption, although left ventricular mass was reported to be increased.

The prevalence of cardiac cachexia in patients with chronic congestive heart failure has been uncertain. However, in a recent study, it was estimated to be 10.5 % in a population of 238 patients with stable congestive heart failure [128]. Importantly, cardiac cachexia in severe heart failure could lead to a number of endocrine disturbances [129], including acquired GH resistance and may explain some of the diverse responses to GH therapy observed in different patients [130].

The reasons for the lack of convincing positive effects of GH on cardiac function in these randomized trials in all likelihood depend upon several factors. Duration of treatment has been relatively short (2–3 months) compared to conventional heart failure trials, and the studies have so far included only a small number of patients, which may also contribute to lack of power and negative results. Encouragingly, a meta-analysis encompassing 12 clinical trials still suggested that GH treatment had beneficial effects on left ventricular geometry, ejection fraction, and exercise parameters, all correlated to increase in serum IGF-I levels [131]. Hence, whether GH treatment will finally find a place in the treatment of heart failure remains to be established and need to be studied in larger placebo-controlled clinical trials.

A topic which has received increased attention during the last years is the endocrine status of heart failure patients. It has been suggested that multiple anabolic deficiencies are common in heart failure patients and that this may be associated with worse outcome [132–134]. At present, the literature is inconsistent regarding the GH/IGF-I axis in heart failure describing both low levels of IGF-I [133, 135, 136] and normal or even higher levels [137]. Possible explanations for these diverging results may be due to heterogeneous heart failure patients and the use of different IGF-I assays. Even less is known about GH secretion in heart failure, although there are indications of disturbed secretion [138]. A more recent study suggests that as many as 40 % may fulfill criteria for GH deficiency [139].

Recently, a novel approach in the selection of heart failure patients to be treated with GH was taken by the group of Saccà and collaborators. Using a GH provocation test, only patients that fulfilled criteria for GH deficiency (*n* = 56) were enrolled in a randomized, single blind study where they were treated with GH for 6 months. Here, GH had a number of beneficial effects compared to controls, including improved quality of life score, increased peak oxygen uptake, exercise duration, and flow-mediated vasodilation. Moreover, GH treatment leads to a moderate but significant increase in left ventricular ejection fraction and a reduction in circulating *N*-terminal pro-brain natriuretic peptide levels [139]. Future studies with more robust RCT design are needed to verify the validity of this approach of selecting heart failure patients for GH. However, preliminarily, results so far are very promising.

## Summary and conclusions

It can be summarized that GH appears to have a number of important effects, both on the heart and the vasculature. Furthermore, an intact GH/IGF-I system with neither deficiency nor excess appears to be optimal for a normal cardiovascular function. Anabolic impairment in heart failure, including possible GH deficiency, opens up interesting perspectives for new treatment modalities with hormones in addition to conventional heart failure in the future.
